# Head and neck cancer of unknown primary: a nationwide analysis of hospital-based cancer registry data in Japan, 2018–2022

**DOI:** 10.1007/s10147-026-02984-6

**Published:** 2026-03-18

**Authors:** Ryoko Rikitake, Yu Mizushima, Toshihiko Sakai, Takahiro Higashi

**Affiliations:** 1 Department of Public Health and Health Policy, Graduate School of Medicine, The University of Tokyo, 7-3-1 Hongo, Bunkyo-Ku, Tokyo, 113-0033 Japan; 2 Department of Head and Neck Surgery, National Cancer Center Hospital, Tokyo, Japan

**Keywords:** Head and neck cancer of unknown primary, Human papillomavirus, Epstein–Barr virus, Oropharyngeal cancer, Epidemiology

## Abstract

**Background:**

Some head and neck cancers are metastatic tumors of unknown primary origin, but their epidemiology has seldom been studied. This study aimed to address this knowledge gap using a nationwide database in Japan. Furthermore, we examined the nationwide implementation of human papillomavirus (HPV) and Epstein–Barr virus (EBV) testing, assessed their inter-facility and temporal variations, and compared these findings with oropharyngeal cancer cases.

**Methods:**

Head and neck squamous cell carcinoma of unknown primary origin and oropharyngeal squamous cell carcinoma diagnosed between 2018 and 2022 were identified from the Hospital-based Cancer Registries of Japan. Data on sex, age, TNM classification, HPV and EBV test results, and treatment facilities were collected. Temporal changes in viral diagnostic practices were analyzed.

**Results:**

We identified 1636 new cases of head and neck cancers of unknown primary. The disease was more common in males and patients aged 70–74 years. HPV positivity was more frequent in patients aged 45–59 years, whereas EBV positivity was less frequent in all age groups. Of the patients, 67.6% were treated at certified head and neck cancer facilities, which had higher viral testing rates than non-certified facilities. Case numbers remained stable over time, whereas viral testing and HPV positivity increased; however, both remained lower than in oropharyngeal cancer cases.

**Conclusions:**

This study visualized real-world virus testing for patients with head and neck cancers of unknown primary, providing insights into diagnostic equity, institutional capacity, and the need for standardized cancer care. Continued surveillance is essential to improve outcomes in this rare condition.

## Introduction

Cancers of unknown primary (CUPs) comprise metastatic cancers whose origin cannot be identified despite standard diagnostic work-ups [1]. They account for 2–5% of new cancers globally [2], with adenocarcinoma being the most common histological type [3]. The incidence is higher in males and older adults. The primary sites are often difficult to detect, but the incidence of CUP has declined, probably due to improved diagnostics [2].

CUPs of the head and neck (HNCUPs) account for 3–9% of all head and neck cancers, most of which are squamous cell carcinomas (SCCs) [4–6]. HNCUPs with SCC refer to metastatic SCCs in the cervical lymph nodes without evidence of a primary site on basic physical examination [7]. Diagnostic imaging studies (including PET/CT), upper gastrointestinal endoscopy with white-light and narrow-band imaging (NBI), and procedures performed under general anesthesia, such as pan-endoscopy and palatine tonsillectomy, are recommended to identify the primary site in cases of HNCUP [8].

In contrast to the overall incidence of CUP, the incidence of HNCUP has increased significantly in the United States in recent years and a high proportion of these cases are human papillomavirus (HPV)–positive. Patients with HPV-positive HNCUPs with SCC tend to be younger males [9]. The primary tumor is typically presumed to originate in the oropharynx when cervical lymph node metastasis is HPV-positive. In contrast, cervical lymph node metastasis associated with Epstein–Barr virus (EBV) is generally considered to originate from the nasopharynx [10]. However, exceptions to these assumptions do exist and if the primary tumor site cannot be identified despite thorough evaluation, the diagnosis should be HNCUP, irrespective of the virological findings obtained from the metastatic cervical lymph nodes.

Identification of the primary tumor site is critical for the management of patients with HNCUPs [11, 12]. Recent advances in molecular and immunohistochemical testing have improved diagnostic precision. However, their implementation in clinical practice varies widely across institutions. The N (nodal) classification tables for patients with HNCUPs have been updated since the 8th edition of the UICC TNM classification was published in 2017. Patients with HPV/p16-positive cervical lymph node metastasis of SCC are classified using the N category for p16-positive oropharyngeal carcinoma, whereas those with EBV-positive cases are assessed according to the N classification for nasopharyngeal carcinoma [13]. However, T classification is T0 (no primary tumor) as the primary tumor is unknown.

The prognostic significance of these viral markers has been highlighted in international reports [14]. The importance of viral tests has also been increasingly recognized. However, data on the frequency of viral marker testing for HNCUP treatment in Japan remain limited. Understanding the distribution of HPV and EBV testing across hospitals is essential to ensure equitable and standardized diagnostic care in Japan, where patients have access to all facilities, including certified head and neck cancer hospitals.

This study aimed to characterize the nationwide implementation of HPV and EBV testing in patients with HNCUPs, examine inter-facility and temporal variations, and contextualize these findings through a comparison with oropharyngeal squamous cell carcinoma (OPSCC) cases using a nationwide database of Hospital-based Cancer Registries (HBCR).

## Patients and methods

### Data source and study population

We analyzed data from the Hospital-based Cancer Registry (HBCR) database of cancer care hospitals designed by the Ministry of Health, Labour, and Welfare in Japan. The database collects standardized clinical and treatment information from more than 700 designated cancer care hospitals. The registry contains demographic variables, cancer site and histology, stage at diagnosis, and diagnostic test results, including HPV (p16-positive immunocytochemistry) and EBV status for head and neck cancer (including lymph nodes with unknown primary). The registry also records the diagnostic basis for cancer cases, including histological examination, cytological examination, and site-specific tumor markers. In cases of HNCUP, the histological examination is based on surgical specimens, and cytological examination is presumed to originate from lymph node samples. However, information regarding whether core needle biopsy was performed is not available, nor is it clear whether histopathological diagnoses were based on lymph node biopsy samples or specimens from neck dissection. The HBCR also records a variable that indicates where the patient started the first course of treatment. Only the cases treated at the registered institutions were included in the analysis.

We identified the patients with HNCUPs diagnosed between January 2018 and December 2022 from the HBCR database based on the International Classification of Diseases for Oncology, 3rd edition (ICD-O-3.2) topography codes (C76.0; lymph node of head and neck, unspecified primary site) and histology codes of the cases with histologically confirmed SCC (ICD-O-3 morphology codes 8070–8078), as SCC is the predominant histological type of HNCUP [4–6]. According to the HBCR, this code (C76.0) specifically represents metastatic lesions in the cervical lymph nodes with an unknown primary site within the head and neck region. We excluded patients with lymphoid diseases, non-epithelial histology, or unknown TNM stages. Different clinical and pathological staging systems were used for CUP based on the results of HPV/EBV testing [13]. HNCUP cases were defined as patients with a histopathological diagnosis of malignancy based on samples obtained from metastatic cervical lymph nodes, but with an unidentified primary tumor site within 1 year of initial cancer diagnosis. The HBCR records the case with the primary site if the investigation identified the primary tumor to be oropharyngeal or nasopharyngeal carcinoma.

We classified the treatment facilities based on the status of the teaching facilities for head and neck surgeons certified by the Japan Society for Head and Neck Surgery. We included the “certified head and neck cancer facilities” as of 2020, including provisionally certified facilities [15].

We also conducted an additional analysis of oropharyngeal cancer (ICD-O-3.2 topography: C01.9, C02.4, C05.1, C05.2, C09.x, C10.0, and C10.2–C10.9) to support the interpretation of the viral status of HNCUPs.

### Data analyses

The Cochran–Armitage trend test was used to examine the trends in virus testing. All database processing and statistical analyses were performed using Stata/MP version 18.1 (StataCorp LLC, College Station, TX, USA).

### Ethical considerations

The study protocol was approved by the Institutional Review Board (IRB) of our institute (2023145NI). Data use was authorized by the Data Utilization Committee of the Hospital-based Cancer Registry of Japan.

Counts less than 10 (excluding zero) were suppressed and reported as “ < 10” in the table to comply with the patient privacy protection rules of cancer registries.

## Results

### Case overview

**Fig. 1:**
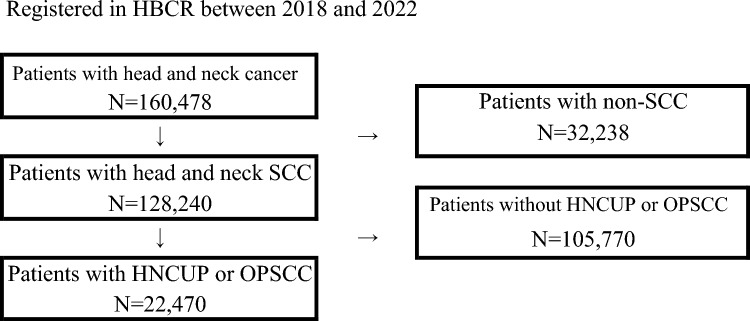
Schematic representation of the study cases

The total number of new cases of head and neck cancers registered in the HBCR between January 2018 and December 2022 was 160,478. Of these, 128,240 were histologically confirmed SCC cases. The numbers of HNCUP and OPSCC cases were 1636 and 20,834, respectively (Fig. 1 and Table 1). These cases were treated at 609 facilities, of which 152 were certified head and neck cancer facilities and 457 were non-certified.

**Table 1:** Characteristics of patients with HNCUP or OPSCC

Variable	Category	N	%
Tumor site			
	HNCUP	1636	7.3%
	OPSCC	20,834	92.7%
Sex			
	Male	18,356	81.7%
	Female	4114	18.3%
Age (years)			
	< 30	< 10	^a^
	30–34	33	0.1%
	35–39	143	0.6%
	40–44	454	2.0%
	45–49	1059	4.7%
	50–54	1571	7.0%
	55–59	2091	9.3%
	60–64	2856	12.7%
	65–69	3892	17.3%
	70–74	4582	20.4%
	75–79	3139	14.0%
	80–84	1690	7.5%
	85–89	729	3.2%
	90–94	184	0.8%
	95–99	37	0.2%
	100 <	< 10	^a^
HPV/EBV testing	performed	18,489	82.3%
	none/unknown, not recorded	3981	17.7%
Facility type	Certified head and neck cancer facility	16,025	71.3%
	Non-certified facility	6445	28.7%
Total		22,470	100%

Less than 10 cases (except for 0) of given age ranges are represented as “ < 10.”

^a^Data could not be disclosed due to patient’s privacy protection

### HNCUPs

HNCUPs were more frequent in males than in females (*N* = 1,310, 80.1%). They were most frequent among patients aged 70–74 years (*N* = 344, 21.0%; Table 2). Cases involving patients younger than 50 years were relatively uncommon (approximately 6%).

**Table 2:** Characteristics of patients and HPV/EBV testing for the HNCUP cases

Variable	Category	N	%	HPV/EBV test performed (N)	HPV/EBV test performed (%)	HPV positive (N)	HPV positive (%)	EBV positive (N)	EBV positive (%)
Sex									
	Male	1,310	80.1%	832	63.5%	320	38.5%	^a^	^a^
	Female	326	19.9%	200	61.3%	88	44.0%	^a^	^a^
Age (years)									
	< 30	0	0%	0	0%	0	0.0%	0	0%
	30–34	< 10	^a^	< 10	66.7%	< 10	50.0%	< 10	50.0%
	35–39	11	0.7%	< 10	^a^	< 10	42.9%	< 10	28.6%
	40–44	22	1.3%	16	72.7%	< 10	^a^	< 10	^a^
	45–49	62	3.8%	47	75.8%	32	68.1%	< 10	^a^
	50–54	108	6.6%	79	73.1%	50	63.3%	0	0%
	55–59	161	9.8%	110	68.3%	59	53.6%	< 10	^a^
	60–64	178	10.9%	122	68.5%	58	47.5%	< 10	^a^
	65–69	311	19.0%	205	65.9%	74	36.1%	< 10	^a^
	70–74	344	21.0%	217	63.1%	61	28.1%	< 10	^a^
	75–79	216	13.2%	123	56.9%	35	28.5%	0	0%
	80–84	140	8.6%	74	52.9%	21	28.4%	< 10	^a^
	85–89	58	3.5%	25	43.1%	< 10	^a^	0	0%
	90–94	14	0.9%	< 10	^a^	< 10	25.0%	< 10	25.0%
	95–99	< 10	^a^	< 10	12.5%	0	0.0%	0	0%
	> 100	0	0%	0	0%	0	0.0%	0	0%
N category									
	N1	507	31.0%	388	76.5%	307	79.1%	11	2.8%
	N2	81	5.0%	77	95.1%	72	93.5%	< 10	^a^
	N2a	127	7.8%	67	52.8%	0	0%	0	0%
	N2b	235	14.4%	114	48.5%	0	0%	0	0%
	N2c	77	4.7%	38	49.4%	0	0%	0	0%
	N3	28	1.7%	28	100%	28	100%	0	0%
	N3a	51	3.1%	23	45.1%	0	0%	0	0%
	N3b	512	31.3%	288	56.3%	0	0%	0	0%
M category									
	M0	1,431	87.5%	920	64.3%	370	40.2%	13	1.4%
	M1	178	10.9%	102	57.3%	37	36.3%	< 10	^a^
UICC stage (8th edition)									
	1	338	20.7%	288	85.2%	288	100.0%	0	0%
	2	78	4.8%	73	93.6%	61	83.6%	10	13.7%
	3	147	9.0%	88	59.9%	20	22.7%	< 10	^a^
	4	38	2.3%	37	97.4%	37	100%	0	0%
	4A	373	22.8%	192	51.5%	0	0%	0	0%
	4B	493	30.1%	278	56.4%	0	0%	< 10	-
	4C	138	8.4%	63	45.7%	0	0%	0	0%
Facility type									
	Certified head and neck cancer facility	1106	67.6%	735	66.5%	290	39.5%	< 10	^a^
	Non-certified facility	530	32.4%	297	56.0%	118	39.7%	< 10	^a^
Total		1,636	100%	1,032	63.1%	408	39.5%	15	1.5%

Less than 10 cases (except for 0) of given sex, age ranges, (T)NM categories, stages, and facility types are represented as “ < 10.”

Cases with Nx,Mx categories or unknown stages were excluded above the table

^a^Data could not be disclosed due to patient's privacy protection

HPV/EBV test results were available for 1032 (63.1%) of the 1,636 HNCUP cases. The results for the remaining cases (36.9%) were missing or unclassified or not available due to non-testing. During the study period, 1465 HNCUP cases (89.5%) were registered as having a histological diagnosis. Among these, 1002 cases (68.3%) underwent HPV and/or EBV viral testing. In addition, a small subset of cases (*N* = 30) had cytological examination as the diagnostic basis, and viral testing was also performed in these cases. Of the 1032 cases with HPV/EBV test results, 408 (39.5%) and 15 (1.5%) were positive for HPV and EBV, respectively. The HPV positivity rate was higher among patients aged 45–59 years. However, the age-related trend in EBV positivity could not be determined due to the limited number of EBV-positive cases (*N* = 15).

The numbers and proportions of the patients in each UICC stage (8th edition of the UICC TNM classification) were as follows: stage I, 338 (20.7%); stage II, 78 (4.8%); stage III, 147 (9.0%); stage IV, 38 (2.3%); stage IVa, 373 (22.8%); stage IVb, 493 (30.1%); and stage IVc, 138 (8.4%).

Of the patients with HNCUP, 67.6% were treated at facilities certified by the Japan Society for Head and Neck Surgery. The HPV/EBV tests were more frequently performed for those diagnosed with HNCUP at the certified head and neck cancer facilities than for those diagnosed at non-certified facilities (66.5% vs 56%, respectively).

**Fig. 2:**
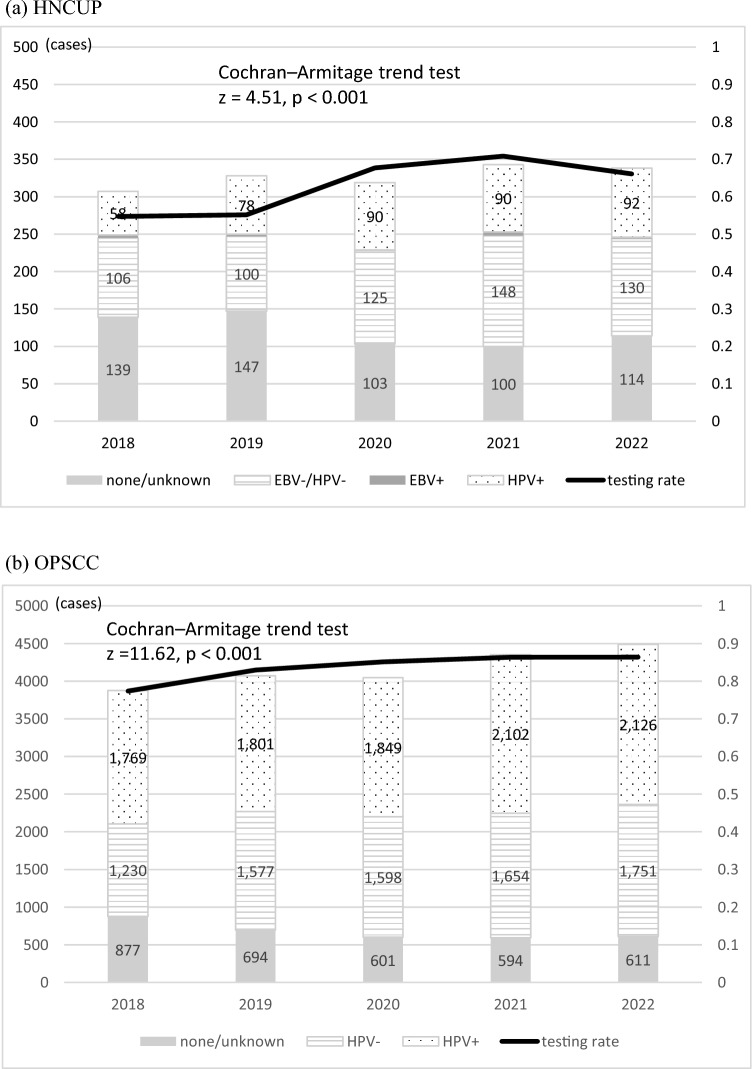
Annual trends in the implementation rate and positivity of virus testing (a) HNCUP. (b) OPSCC

The number of registered HNCUP cases during the study period remained stable, ranging from 307 to 343 cases annually. The HPV/EBV testing rate increased from 54.7% in 2018 to 66.1% in 2022 (Cochran–Armitage trend test, z = 4.51, *p* < 0.001). The proportion of HPV-positive cases (HPV positivity rate) increased from 34.5% in 2018 to 40.9% in 2022 (Fig. 2).

### OPSCC

For OPSCC, 81.8% (*N* = 17,046) were males, and the most frequent age group was 70–74 years (*N* = 4238, 20.3%, Table 3). The distribution of patients aged 45–49 years was more continuous for those with OPSCC than for those with HNCUP, indicating a relatively higher proportion of younger individuals in the former cohort.

**Table 3:** Characteristics of patients and HPV testing in those with OPSCC

Variable	Category	N	%	HPV test performed (N)	HPV test performed (%)	HPV positive (N)	HPV positive (%)
Sex							
	Male	17,046	81.8%	14,205	83.3%	7,594	53.5%
	Female	3788	18.2%	3252	85.9%	2,053	63.1%
Age (years)							
	< 30	< 10	^a^	< 10	50.0%	< 10	50.0%
	30–34	30	0.1%	23	76.7%	14	60.9%
	35–39	132	0.6%	118	89.4%	99	83.9%
	40–44	432	2.1%	379	87.7%	307	81.0%
	45–49	997	4.8%	889	89.2%	693	78.0%
	50–54	1463	7.0%	1283	87.7%	939	73.2%
	55–59	1930	9.3%	1664	86.2%	1075	64.6%
	60–64	2678	12.9%	2257	84.3%	1281	56.8%
	65–69	3581	17.2%	3012	84.1%	1547	51.4%
	70–74	4238	20.3%	3524	83.2%	1727	49.0%
	75–79	2923	14.0%	2411	82.5%	1114	46.2%
	80–84	1550	7.4%	1253	80.8%	577	46.0%
	85–89	671	3.2%	501	74.7%	207	41.3%
	90–94	170	0.8%	119	70.0%	57	47.9%
	95–99	29	0.1%	18	62.1%	< 10	^a^
	> 100	< 10	^a^	< 10	100%	< 10	50.0%
T category							
	T0	24	0.1%	20	83.3%	20	100%
	Tis	1154	5.5%	502	43.5%	64	12.7%
	T1	4516	21.7%	3361	74.4%	1774	52.8%
	T2	7674	36.8%	6961	90.7%	4473	64.3%
	T3	3079	14.8%	2806	91.1%	1399	49.9%
	T4	1802	8.6%	1796	99.7%	1794	99.9%
	T4a	1714	8.2%	1403	81.9%	< 10	–
	T4b	463	2.2%	372	80.3%	< 10	–
	TX	408	2.0%	236	57.8%	119	50.4%
N category	N0	7,708	37.0%	5471	71.0%	1887	34.5%
	N1	6545	31.4%	6171	94.3%	5369	87.0%
	N2	1981	9.5%	1949	98.4%	1,945	99.8%
	N2a	198	1.0%	151	76.3%	< 10	–
	N2b	1470	7.1%	1245	84.7%	< 10	–
	N2c	1200	5.8%	1005	83.8%	0	0%
	N3	386	1.9%	386	100.0%	386	100%
	N3a	59	0.3%	40	67.8%	0	0%
	N3b	1072	5.1%	935	87.2%	< 10	-
	NX	215	1.0%	104	48.4%	47	45.2%
M category							
	M0	19,845	95.3%	16,675	84.0%	9269	55.6%
	M1	788	3.8%	680	86.3%	332	48.8%
	MX	201	1.0%	102	50.7%	46	45.1%
UICC stage (8th edition)							
	0	1159	5.6%	505	43.6%	64	12.7%
	1	7479	35.9%	6,363	85.1%	5173	81.3%
	2	3753	18.0%	3,331	88.8%	2148	64.5%
	3	3063	14.7%	2,821	92.1%	1,807	64.1%
	4	337	1.6%	333	98.8%	332	99.7%
	4A	2967	14.2%	2,489	83.9%	< 10	-
	4B	1251	6.0%	1,072	85.7%	< 10	-
	4C	451	2.2%	347	76.9%	0	0%
	unknown	374	1.8%	196	52.4%	112	57.1%
Facility type							
	Certified head and neck cancer facility	14,919	71.6%	12,784	85.7%	7035	55.0%
	Non-certified facility	5915	28.4%	4,673	79.0%	2612	55.9%
Total		20,834	100%	17,457	83.8%	9647	55.3%

Less than 10 cases (except for 0) of give age ranges, TN(M) categories, and stages are represented as “ < 10.”

^a^Data could not be disclosed due to patient's privacy protection

Of the 20,834 patients with OPSCC, 17,457 cases (83.8%) underwent HPV testing. HPV testing was conducted for 83.3% and 85.9% of the male and female patients with OPSCC, respectively. Of the 17,457 cases tested, 9,647 (55.3%) were positive for HPV. Viral tests were performed for 85.7% of the patients with OPSCC at the certified head and neck cancer facilities.

The number of OPSCC cases increased from 3876 to 4488 during the study period. The HPV testing rate increased from 77.4% in 2018 to 86.4% in 2022 (Cochran–Armitage trend test, z = 11.62, *p* < 0.001). The proportions of HPV-positive cases (HPV positivity rates) were relatively stable during the study period, ranging from 53.6 to 59% (Fig. 2).

## Discussion

This study used HBCR data to elucidate the epidemiological characteristics of HNCUP in Japan. The comprehensive scope of the HBCR offers a unique opportunity to evaluate national diagnostic practices for HNCUP, with a focus on HPV/EBV testing. This testing is widely acknowledged to be crucial for accurate diagnosis and treatment planning, including the determination of radiation fields [7]. Benchmarking against OPSCC cases facilitates the contextualization of HPV positivity among HNCUP cases and strengthens the understanding of viral oncogenesis patterns of head and neck cancers. Discrepancies in the implementation of HPV/EBV testing persist, likely reflecting variations in diagnostic resources, institutional expertise, and clinician awareness [16].

HNCUP was more prevalent among males and individuals aged 70–74 years. The predominance of males and older adults is consistent with the general epidemiological patterns observed for head and neck cancers. The incidence of HPV positivity was higher in patients with HNCUPs aged 45–59 years, which was consistent with OPSCC observations. This finding indicates that HPV testing may provide valuable insights into the accurate diagnosis and localization of the primary site in younger cohorts of patients with HNCUPs.

Only 15 cases (1.5%) were positive for EBV in this study. According to the New Classification of Rare Cancers (NCRC) system, the average annual incidence rate of nasopharyngeal cancer in Japan was calculated as 0.78 per 100,000 population [17]. The epidemiology of EBV among patients with HNCUP is expected to be elucidated, as more cases are reported in the future.

The prevalence of HPV/EBV testing among patients diagnosed with HNCUP was higher at the certified head and neck cancer facilities than that at non-certified facilities (66.5% vs. 56%). More than 60% of head and neck cancer treatments in Japan are conducted at certified teaching facilities [18]. The Japan Society for Head and Neck Surgery established a framework for specialist certification and institutional accreditation to improve the diagnosis and treatment of head and neck cancers and reduce the need for inter-institutional care. Our study revealed that these certified facilities conducted viral testing more frequently, in accordance with recent clinical guidelines. This observation suggests that the degree of viral testing implementation may be contingent on the level of development or resources available within the institutional clinical infrastructure. Future research should explore the factors that serve as barriers to HPV/EBV testing.

The incidence of registered HNCUP has remained relatively stable over the years. However, the rates of HPV and EBV testing and their positivity have increased. The rates of HPV and EBV testing increased from 54.7% in 2018 to 66.1% in 2022. However, these rates were lower than the 83.8% HPV testing rate for OPSCC cases. Although a small subset of cases (N = 30) in our study had cytological examination as the diagnostic basis, with viral testing also performed, the lower rate of HPV/EBV testing in HNCUP compared with OPSCC may partly be attributable to the requirement for tissue sampling from cervical lymph nodes for viral testing [19]. Some institutions may still hesitate to perform needle biopsy or lymph node excision of the cervical nodes due to concerns about potential tumor dissemination and procedural invasiveness for patients. The ideal treatment for HNCUP is still debated [20]. However, identifying the primary tumor is an important step toward better management of patients with HNCUP. Even when the primary site remains unknown, HPV and EBV testing may assist in selecting appropriate treatment strategies, determining treatment intensity, and guiding follow-up. Testing for HPV and EBV is beneficial for this purpose and should be incorporated into the standard diagnostic work-up.

The HPV positivity rate increased from 34.5% in 2018 to 40.9% in 2022. This trend suggests a potential increase in cases with latent HPV-positive oropharyngeal primaries among patients with HNCUP, highlighting an important area for future investigation. A recent report highlighted the increased detection of oropharyngeal primary tumors and the association of HPV positivity with higher odds of OPSCC detection [21]. Diagnostic transoral robotic surgery (TORS) has emerged as an important modality for identifying elusive primary sites, especially in patients with HPV-positive HNCUP. Notably, TORS facilitates comprehensive evaluation of the oropharynx, including the base of tongue, and has been reported to achieve primary tumor identification rates exceeding 70% [22].

This study has some limitations. First, the study period was relatively short. Further case accumulation and longer-term studies are required to elucidate the precise epidemiology of HNCUP, especially EBV-mediated rare entities. Second, the HBCR in Japan does not include detailed data on specific diagnostic procedures or treatments performed for each case. For example, although surgery may be recorded as having been performed, the exact surgical approach—such as curative resection or biopsy—cannot be determined. Third, comprehensive long-term clinical course and definitive prognostic data for patients with HNCUP are unavailable. The eventual identification of the primary tumor is critically important. The date of cancer diagnosis typically serves as the reference point for determining the time of diagnosis in the HBCR, as exemplified by OPSCC. In contrast, HNCUP is definitively diagnosed when no primary tumor is detected after a period of thorough investigation to identify a primary tumor site. Even when HPV or EBV testing yields a positive result, the diagnosis remains classified as CUP if no primary tumor has been identified at the time of cancer registration. This approach is consistent with the 8th edition of the UICC classification and current clinical practice guidelines for head and neck cancer. The study population likely included cases of p16-positive oropharyngeal carcinoma, EBV-associated nasopharyngeal carcinoma, or cancers originating from other primary sites (such as oral cancer, metastases from subclavicular organs, and thyroid cancer) in patients who tested negative for both HPV and EBV after long-term follow-up of tumor progression. Finally, it is currently impractical to accurately estimate the nationwide incidence of HNCUP, given that the HBCR coverage rate is approximately 70% [23]. Therefore, documenting HNCUP cases based on standardized diagnostic criteria within cancer registries that encompass the entire Japanese population, such as the National Cancer Registry, will make accurate estimation of the incidence of the disease feasible.

## Conclusions

This study used a nationwide database in Japan to thoroughly characterize the epidemiology of HNCUP in a sufficiently large patient cohort. HNCUP is rare in Japan, and ongoing efforts to monitor it are crucial for enhancing the medical care of affected patients. This study elucidated the real-world implementation of HPV and EBV testing for HNCUP. Viral testing is clinically recommended for the diagnosis of HNCUP, particularly in identifying the primary lesion. Comparison between HNCUP and OPSCC revealed that, although the implementation rate of viral testing in HNCUP remained lower than that in OPSCC, a clear increasing trend was observed among HNCUP cases. This finding suggests that recommended diagnostic tests are being increasingly adopted in routine clinical practice in Japan, with particularly higher implementation rates at certified head and neck cancer facilities. These findings provide significant insights into diagnostic equity, institutional capacity, and future policy discussions concerning standardization strategies and the development of performance indicators within the cancer care system of Japan.

## Data Availability

The analysis used individual-level data from the Hospital-based Cancer Registry in Japan, which is not publicly available because of legal and privacy restrictions. Access may be granted with approval from the Committee of the Hospital-based Cancer Registry Office.
